# Development and Management of Networks of Care at the End of Life (the REDCUIDA Intervention): Protocol for a Nonrandomized Controlled Trial

**DOI:** 10.2196/10515

**Published:** 2018-10-12

**Authors:** Silvia Librada Flores, Emilio Herrera Molina, Fátima Díaz Díez, María José Redondo Moralo, Cristina Castillo Rodríguez, Kathleen McLoughlin, Julian Abel, Tamen Jadad Garcia, Miguel Ángel Lucas Díaz, Inmaculada Trabado Lara, María Dolores Guerra-Martín, María Nabal

**Affiliations:** 1 New Health Foundation Seville Spain; 2 Palliative Care Team Extremaduran Health Service Badajoz Spain; 3 Department of General Practice University College Cork Ireland; 4 Department of Palliative Care Weston Area Health Trust Weston-super-Mare United Kingdom; 5 Department of Nursing University of Seville Seville Spain; 6 Palliative Care Team Hospital Arnau de Vilanova Lleida Spain

**Keywords:** palliative care, public health, delivery of health care, community networks

## Abstract

**Background:**

End-of-life needs can be only partly met by formalized health and palliative care resources. This creates the opportunity for the social support network of family and community to play a crucial role in this stage of life. Compassionate communities can be the missing piece to a complete care model at the end of life.

**Objective:**

The main objective of this study is to evaluate the REDCUIDA (*Redes de Cuidados* or Network of Care) intervention for the development and management of networks of care around people with advanced disease or at the end of life.

**Methods:**

The study is a 2-year nonrandomized controlled trial using 2 parallel groups. For the intervention group, we will combine palliative care treatment with a community promoter intervention, compared with a control group without intervention. Participants will be patients under a community palliative care team’s supervision with and without intervention. The community promotor will deliver the intervention in 7 sessions at 2 levels: the patient and family level will identify unmet needs, and the community level will activate resources to develop social networks to satisfy patient and family needs. A sample size of 320 patients per group per 100,000 inhabitants will offer adequate information and will give the study 80% power to detect a 20% increase in unmet needs, decrease families’ burden, improve families’ satisfaction, and decrease the use of health system resources, the primary end point. Results will be based on patients’ baseline and final analysis (after 7 weeks of the intervention). We will carry out descriptive analyses of variables related to patients’ needs and of people involved in the social network. We will analyze pre- and postintervention data for each group, including measures of central tendency, confidence intervals for the 95% average, contingency tables, and a linear regression. For continuous variables, we will use Student *t* test to compare independent samples with normal distribution and Mann-Whitney *U* test for nonnormal distributions. For discrete variables, we will use Mann-Whitney *U* test. For dichotomous variables we will use Pearson chi-square test. All tests will be carried out with a significance level alpha=.05.

**Results:**

Ethical approval for this study was given by the Clinical Research Committee of Andalusian Health Service, Spain (CI 1020-N-17), in June 2018. The community promoter has been identified, received an expert community-based palliative care course, and will start making contacts in the community and the palliative care teams involved in the research project.

**Conclusions:**

The results of this study will provide evidence of the benefit of the REDCUIDA protocol on the development and assessment of networks of compassionate communities at the end of life. It will provide information about clinical and emotional improvements, satisfaction, proxy burden, and health care resource consumption regarding patients in palliative care.

**Registered Report Identifier:**

RR1-10.2196/10515

## Introduction

### Palliative Care in the Community

Palliative care provides professional, scientific, and human responses to the needs of those living with advanced disease or facing the end of life, while also supporting their families [1]. Experts in this field are organized into multidisciplinary teams to provide a comprehensive care model to address suffering, symptom management, and other emotional, social, and spiritual aspects of the final stage of life, death itself, and the grief of relatives [2]. Palliative care is a type of care that best responds to the needs of these people and their families in such circumstances [3] and is internationally recognized as a right of citizens [4]. Palliative care can be provided across multiple settings. Studies indicate that, while aiming to be holistic, palliative care resources cannot possibly cover all patient and family needs [5]. Furthermore, differences in family structure mean that some patients might require more social and practical support than others. In these cases, mobilization of a person’s wider support networks can play a crucial role [6].

The main family caregiver is recognized as the person most involved in the patient’s care. This person, in Latin countries, usually is the contact with health and social care professionals and has to cope with the patient’s daily physical, social, and emotional needs [7]. Outside of the immediate family network, other support networks can assume other necessary tasks [6]. Evidence suggests that up to 7 different profiles have been identified of people who participate in the care of someone facing a terminal illness [8]. Mobilization of this wider network is the base of compassionate communities [9].

Kellehear’s theoretical framework of a compassionate community is gathering momentum internationally [10-12]. This new model of care operating in countries including the United Kingdom [13], Ireland [14], India [15], Canada [16], Australia [17,18], and Spain [19,20] brings together not only health and social professionals and primary caregivers but also the wider community (including extended family members, friends, neighbors, volunteers, and work colleagues) to support people and their families at the end of life. At a wider organizational level, the model of care may also include schools, universities, workplaces, companies, the arts community, social care and community development organizations, and policy makers [21]. It is intended that, through such a model of community intervention, there is an awakening and heightened activity of citizens regarding palliative and compassionate care [10-22]. Compassionate communities integrate and promote palliative care socially [23-25].

Health care organizations and policy makers are increasingly involved in the design, development, and evaluation of compassionate community models. It is recognized that they offer an opportunity to support the reconfiguration of health and social services, reduce costs, and facilitate models of integrated care [9]. The World Health Organization has included the development of compassionate communities based on awareness, training, and implementation of networks into their guide for the planning and implementation of palliative care services [26,27].

One of the largest and most successful models of compassionate communities in the world began in Seville, Spain. All with You [28] (a direct translation of the program’s Spanish name, *Todos Contigo*) is a social innovation program created by the New Health Foundation in 2014 [19]. It seeks to transform care for people with advanced chronic conditions that require palliative care by monitoring and optimizing health care and social services, providing support to families, mobilizing community-based assets, and promoting greater awareness of the challenges and opportunities associated with palliative care and the management of complex chronic conditions.

To our knowledge, no protocol or tool is available to assess systematically these types of intervention, so it remains difficult to compare them and to assess their real effect.

As a part of the All with You program, the REDCUIDA (short for *Redes de Cuidados*, or Network of Care) intervention protocol has been developed. This protocol will offer a systematic method to assess the quality of the community intervention and its effect on clinical well-being, patient and family satisfaction, and consumption of health system resources.

### Objective

The primary objective of this study is to evaluate the REDCUIDA intervention protocol for the creation and management of networks of care that cover the unmet needs of a person with advanced diseases or at the end of life.

The secondary objectives are to (1) identify the precise nature of a patient’s unmet needs by palliative care teams that can be addressed through mobilization of the community, (2) detect members of the support networks that can meet the patient’s identified needs and describe their fundamental characteristics (caregiver profiles), (3) analyze the influence of a community promoter’s interventions on the emergence and growth of support networks as the disease progresses, (4) assess whether the REDCUIDA intervention improves the patient’s quality of life and decreases the main caregiver’s burden, (5) establish whether this intervention reduces professionals’ workload and health and social resource consumption during end-of-life care, and (6) analyze the influence of the REDCUIDA intervention on the preference of the place of care and death.

### Hypothesis

We hypothesize that the use of the REDCUIDA protocol in a community intervention program allows for the expansion of care networks that can meet the needs and improve the quality of life of patients, increase family satisfaction, reduce the burden on main caregivers, improve the possibilities of care and death in the preferred place, and reduce the consumption of health care and social care resources during the end of life.

## Methods

### Trial Design

The study is a 2-year nonrandomized controlled trial using a 2-arm parallel group design conducted in the community. For the intervention group, we will combine the standard palliative care treatment with a community promoter’s intervention; the control group will benefit from standard palliative care.

Intervention and control group participants will be patients under the community palliative care team’s supervision living in 2 areas with and without community promoter intervention.

### Setting

The study will be developed in the community, in 2 different geographic areas in Seville, Spain. The main difference between them will be the presence of a community promoter as a part of a new city program called All with You [19].

### Eligibility Criteria

Inclusion criteria are patients living in Seville, with any advanced or terminal illness and receiving palliative care supervision, and having any of the following conditions: (1) total or serious dependence for basic and instrumental daily activities (Barthel Activities of Daily Living Index score <40; Lawton and Brody Instrumental Activities of Daily Living Scale score <3), (2) more than 40% of their needs not covered by the community, (3) a high score in loneliness on the Scale of Social Loneliness (ESTE) II (score >20 points), (4) having a person (family member, friend, neighbor, social worker, or other person) who acts as a communicator and principal person for support and is prepared and able to participate in the development of the network of care and share information with the community promoter, (5) having a main caregiver with an intense physical or emotional burden (scoring >56 on the Zarit Scale), and (6) accepting of the support and guidance by the community promoter with informed consent.

Exclusion criteria are patients who are in a very advanced terminal stage with life expectancy less than 1 week; those who do not have a high level of dependency and have their needs met by their family and other members of the community; and those who do not wish to participate.

### Recruitment Procedure

Potential participants will receive information from palliative care team members. Reasons for declining to participate will be recorded as not interested, too busy, don’t believe in it, and other.

Palliative care teams will arrange an appointment with the patient for the baseline assessment. This first appointment will take place in the patient’s home. At this appointment, written informed consent for participation in the study will be obtained from participants.

Patients assigned to the intervention group will be those living in the San Pablo-Santa Justa area, Seville (60,000 inhabitants), who accepted the community promoter intervention. Patients assigned to the control group will be those living in an area with no community promoter available.

### Intervention

**Figure 1:**
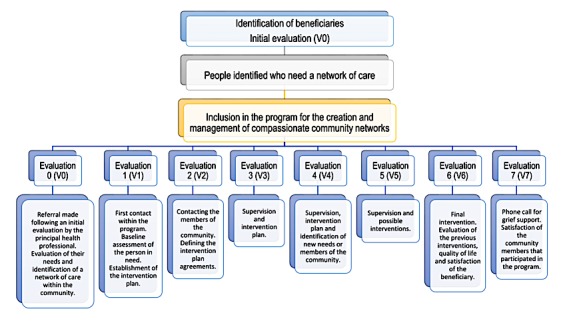
Action procedures for the REDCUIDA protocol.

We will deliver the REDCUIDA intervention over the course of 7 weeks (Figure 1). Different interventions will take place each week during a face-to-face meeting between the community promoter and the person living with advanced illness, or their families, or both.

We will conduct an initial (V0) assessment of the sociodemographic data of the beneficiary and his or her needs. This first step aims to detect the degree of care and support networks that could be mobilized during the progression of the disease. At this point, the community promoter will complete the beneficiary’s referral sheet (Multimedia Appendix 1) and the requirements sheet (Multimedia Appendix 2 [29]). If more detailed information will be needed, a face-to-face meeting will be held with the health professional involved.

Following V0, we will determine the needs of care networks and interventions. Then, we will arrange a meeting among the beneficiary and his or her family, health care professionals, and the community promoter to inform all of them about creating and managing a specific caring network within their community (V1). This first visit will be used to understand the starting point as a baseline analysis for the activities ahead.

During the subsequent interventions, we will conduct several assessments (Table 1) based on the following scales. We will use the Barthel Index [29], in the original or a validated Spanish version [30], to identify needs related to basic daily living activities. This assessment can be self-administered, evaluated with direct observation, or completed by the patient or caregivers (Multimedia Appendix 2). We will use Lawton and Brody’s original scale [31] to identify needs for instrumental daily living activities. This assessment can be self-administered, directly observed, or completed by the patient or caregivers (Multimedia Appendix 2).

**Table 1:** REDCUIDA protocol for applying assessments to the beneficiary and the networks of care.

Assessment	Evaluation step							
	V0	V1	V2	V3	V4	V5	V6	V7
Inclusion and referral sheet	Yes	—	—	—	—	—	—	—
Networks of care	Yes	Yes	Yes	Yes	Yes	Yes	Yes	—
Barthel Index	Yes	Yes	Yes	Yes	Yes	Yes	Yes	—
Lawton and Brody scale	Yes	Yes	Yes	Yes	Yes	Yes	Yes	—
Loneliness scale	Yes	—	—	—	—	—	Yes	—
Zarit Scale	Yes	—	—	—	—	—	Yes	—
EQ-5D-3L^a^	—	—	—	—	—	—	Yes	—
Family and network of care satisfaction	—	—	—	—	—	—	—	Yes

^a^EQ-5D-3L: 3-level EuroQol 5 dimensions questionnaire.

We have adapted Abel and colleagues’ circles of care (Multimedia Appendix 3) [6], which can be self-administered or assessed via a direct interview with the patient or their caregivers. We will detect social solitude using the expanded ESTE II Scale [32] adapted from De Jong Gierveld and Van Tilburg [33], which must be administered directly to the patient or by the caregiver (Multimedia Appendix 4). We will use the 3-level EuroQol 5 dimensions questionnaire (EQ-5D-3L) [34] to asses quality of life. This descriptive system contains 5 dimensions of health (mobility, personal care, daily activities, pain and discomfort, and anxiety and depression), and each has 3 levels of severity (no problems, some problems or moderate problems, and serious problems). This assessment is self-administered or completed directly by the patient (Multimedia Appendix 5). We will use the Zarit Scale [35,36] adapted into Spanish [37] to assess the variable burden of the caregiver in the provision of care to chronically ill patients, which is administered by directly interviewing the caregiver (Multimedia Appendix 6). The Satisfaction Scale, adapted from Villavicencio et al [38] and Molina et al [39] (Multimedia Appendix 7), will be conducted by an external professional by phone on completion of the interventions.

During the intervention processes and the creation of networks of care, the community promoter will follow a series of activities based on Horsfall and colleagues’ method for the creation of ecosystems of care around people at the end of life [40].

At the end of the interventions, a final evaluation (V7) will be conducted to reevaluate the needs coverage by the network and to evaluate family and caregivers’ satisfaction. This phone questionnaire will be administered by an independent professional to avoid information bias.

### Variables

The variables we will use for the descriptive study on the REDCUIDA protocol are the following: for patients’ clinical and sociodemographic data: age, sex, and diagnosis; for families’ sociodemographic data: age, sex, and relationship to the patient; for network of care profiles: relationship to the patient, age, and sex; number of needs according to Barthel Index and the Lawton and Brody scale; number of members of the care network; number of needs covered by the network; quality of life according to the quality of life scale EQ-5D-3L; degree of loneliness according to ESTE II Scale; burden of the principal caregiver according to the Zarit Scale; satisfaction regarding the care network according to the Satisfaction Scale; and place of death.

For community promoter activity data, we will determine the number of interventions performed at home and number of interventions carried out in the community (eg, district, neighborhood, city, community of neighbors).

To assess health care system resources, we will determine the number of hospital admissions in the last month; days of hospital stays in the last month; number of emergency visits in the last month; number of visits of the palliative care team to the home; number of visits of the palliative care team to a hospital; and number of telephone calls made by the palliative care team.

### Sample Size Calculation

We have calculated a sample size of 320 patients per group for a population of 100,000 inhabitants based on the number of people dying of cancer each year (7000 people per million inhabitants) and the number of people in the final stage of nononcologic illnesses (approximately 4500 cases per million inhabitant). In a population of about 700 people, per year, 65% will require specialized palliative care (455 patients eligible for the study [41]).

We have considered that 30% of patients will not sign the informed consent forms or will not meet all the inclusion and exclusion criteria.

### Sources of Information and Data Collection

We will collect data from the patient’s own medical history and the information from the REDCUIDA protocol.

Data regarding variables not included in the protocol, such as the use of the health care system, will be collected through a direct interview with the patient or caregiver and the principal health care professionals regarding visits in the last month (Multimedia Appendix 8). In addition, we will ask palliative care professionals for these data (before and at the end of the care process).

The community promoter and health care professionals will have access to the beneficiary’s clinical information once enrolled in a palliative care program. The permission of the beneficiary and their main caregiver or closest connection shall be required in writing and verbally in order to be able to access the data and use the corresponding data of the interventions for analytical purposes. To ensure confidentiality, the beneficiary’s identification data will be coded so that they can’t be identified by their clinical information.

The deidentified data will be returned to the community promoter and the New Health Foundation for data processing and analysis. The questionnaires will be coded with an alphanumeric identifier in a separate database independent of that containing the participant’s identification data.

### Statistical Methods

We will carry out an initial descriptive analysis of variables related to patients’ needs and the profiles of people involved in the social network by degree of kinship.

We will analyze pre- and postintervention data for each group. These will include measures of central tendency (mean), confidence intervals for the 95% average, contingency tables (frequencies), and a linear regression.

To compare the groups, we will compare means. For continuous variables, we will use Student *t* test to compare independent samples with normal distribution and Mann-Whitney *U* test for nonnormal distributions. For discrete variables, we will use Mann-Whitney *U* test. For dichotomous variables (comparison of proportions), we will use Pearson chi-square test. All tests will be carried out with a significance level alpha=.05.

### Ethical Considerations

Ethical approval for this study was given by the Clinical Research Committee of Andalusian Health Service, Spain (CI 1020-N-17), in June 2018. The study uses informed consent sheets approved by the Clinical Research Committee of Andalusian Health Service, Spain. The right to guarantee data protection will be fulfilled.

## Results

This is a 2-year nonrandomized trial. The protocol has been approved by the Clinical Research Committee of Andalusian Health Service. The community promoter has been identified and has received an expert community-based palliative care course (550 hours). The community promoter will start making contacts in the community and the palliative care teams involved in the research project.

## Discussion

### Beneficiary Population

Results from this study would be applicable among a vast population, including palliative care patients in developing countries. It is known that 7000 people per million population globally die each year, 2500 per million die of cancer, and approximately 4500 per million die in the final stage of any nononcologic illnesses [41]. It is estimated that 65% of this population will need specialized palliative care.

If our results confirm that community social networks improve patients’ and families’ satisfaction at the end of life and correlates with the best use of health system resources, new palliative care systems may be developed.

### Possible Limitations of the Study

As it is not a randomized clinical trial, some selection bias could be considered. If the protocol offers positive results, further randomized investigations will be warranted.

It could be difficult to achieve a clear conclusion if the intervention and control group results are very different, although our results from the descriptive analysis of the intervention group will provide relevant information.

We acknowledge that other clinical or psychosocial variables not included in this protocol can influence patients’ needs and satisfaction. We have chosen basic variables that are recorded in the patient’s medical history and are part of the palliative care approach. Depending on the results, we will consider modifying them for future studies in this line of research.

### Conclusions

This is, to our knowledge, one of the first trials to measure the effectiveness of a nonprofessional network intervention on patient and family satisfaction, family burden, and use of heath resources.

The results of this study may provide some directions for future palliative care interventions at the community level with frail populations. These interventions may also provide a basis for training health professionals and social resources to improve patient–professional communication about end-of-life care for patients at home and stimulate the development of systematic palliative care community networks for this population.

## Appendix

Multimedia Appendix 1 REDCUIDA protocol: inclusion and referral of the beneficiary.

Multimedia Appendix 2 REDCUIDA protocol: beneficiary's scale of needs (adapted from Mahoney and Barthel [29]).

Multimedia Appendix 3 Circle of the community network.

Multimedia Appendix 4 ESTE II Loneliness Scale.

Multimedia Appendix 5 EQ-5D-3L quality-of-life scale.

Multimedia Appendix 6 Zarit Scale for caregiver burden.

Multimedia Appendix 7 Family and social support network scale of satisfaction.

Multimedia Appendix 8 Questionnaire on use of the health care system.

## Abbreviations

EQ-5D-3L: 3-level EuroQol 5 dimensions questionnaire
ESTE II: Scale of Social Loneliness II
REDCUIDA: *Redes de Cuidados* (Network of Care)
