# Supplementary material for: Development and Management of Networks of Care at the End of Life (the REDCUIDA Intervention): Protocol for a Nonrandomized Controlled Trial
Source: JMIR Res Protoc. 2018 Oct 12;7(10):e10515. doi: 10.2196/10515 (PMC6231747; doi:10.2196/10515)
Supplement: Multimedia Appendix 1 [file resprot_v7i10e10515_app1.pdf]

Appendix 1. REDCUIDA PROTOCOL: INCLUSION AND REFERRAL OF THE BENEFICIARY

Criteria for inclusion

- A person with advanced disease or at the end of life that is receiving care from professionals of a palliative care team, residence, hospice or other service for end of life care, that have any of the following characteristics:
    - Total or serious dependence in terms of basic and instrumental daily activities (Barthel Index <40; Lawton and Brody Index <3).
    - More than 40% of their needs not covered by the community.
    - A high score in terms of loneliness (Points on the ESTE II Scale > 20).
    - Have a person (family member, friend, neighbour, social worker or other figure) who acts as a communicator and principal person for support and is prepared and able to participate in the development of the network of care and share information with the community promoter.
    - The main caregiver has an intense physical or emotional burden (Scoring > 56 on the Zarit scale).
- That they accept the support and guidance by the community promoter with informed consent.

Criteria for exclusion:

- Those who are in a very advanced terminal stage with life expectancy below 1 week.
- Those who do not have high level of dependency and have their needs met by their family and other members of the community.
- Those who do not wish to participate in the program.

NOTIFICATION DATE:
DATE OF THE FIRST VISIT:

DATE AND REASON FOR LEAVING THE STUDY:
PLACE OF CARE/ DEATH:

- Hospital
- Home
- Residential centre
- Hospice
- Other

BENEFICIARY'S DETAILS	
Full name:	
Date of Birth/ Age:	
Address:	
Phone number:	
Identification number:	
Clinical history number:	

Oncological or non-oncological diagnosis:
Treatments:
Allergies:
Spiritual needs/beliefs: No Yes
Which ones?
Hobbies:
Occupation:

RATING OF THE LIVING SPACE (Please complete if applicable)

	**Yes**	**No**		**Yes**	**No**		**Yes**	**No**		**Yes**	**No**
Owned			Running water			Barriers			Heating		
Telephone			Elevator			Bathroom/Shower			Cleanliness		

Available systems adapted for the disease / technical aids: adjustable bed, sidebars, chair, adapted toilet, technical aids.

Others:
Referral details:
Name of the centre (Healthcare centre, hospital, and residence/ day centre):
Healthcare professional referrer:
Referring doctor:
Case managing nurse:
Social worker:
Other professional:
Contact:
Community contribution:
Referring person:
Relationship with the beneficiary:
Contact:

**DETAILS OF THE SOCIAL SUPPORT NETWORK**	
**Social support network:**	Only family based: Yes No
**Social worker evaluation:**	Hospital care:<br>Social services:<br>Primary care:
**Social care resources**	Home care service<br>Volunteering<br>Dependency request<br>Tele-assistance<br>Day Centre / Residence
**Social and health care resources**	Public:<br>Private:<br>Mixed:
**DETAILS OF THE MAIN CAREGIVER AND OTHER SUPPORT NETWORKS**<br>**(To be completed by each person identified within the community)**	
**Full name:**	
**Date of birth/ age:**	
**Address:**	Same home. Other:
**Phone number:**	
**Identification number:**	
**Occupation:**	
**Illness/Diagnosis:**	
**Relationship with the patient**	Family member:<br>Father Mother Daughter/son Sibling<br>Spouse Niece/Nephew Grandchild Daughter/son-in-law<br>Non-family member<br>Friend Neighbour Professional caregiver<br>Volunteer Employee Professional colleague<br>Principal caregiver Legal advisor<br>Other:
**Level of involvement**	Excessive High Low Not involved<br>Number of hours daily:
**Instruction of care comprehension**	High:<br>Medium:<br>Low:
**Risk of high burden**	Emotional:<br>Physical:

	No risk detected:
