# Supplementary material for: Development and Management of Networks of Care at the End of Life (the REDCUIDA Intervention): Protocol for a Nonrandomized Controlled Trial
Source: JMIR Res Protoc. 2018 Oct 12;7(10):e10515. doi: 10.2196/10515 (PMC6231747; doi:10.2196/10515)
Supplement: Multimedia Appendix 2 [file resprot_v7i10e10515_app2.pdf]

APPENDIX 2. REDCUIDA PROTOCOL: BENEFICIARY'S SCALE OF NEEDS (Adapted from Barthel, Lawton and Brody)

At each assessment the identification of the needs met or not by the network will be evaluated. As the interventions are conducted the needs scales should be included in the profile of the person who will be able to satisfy the previously unmet needs.

NEEDS (Adapted from Barthel, Lawton and Brody)	SATISFIED								In the case of marking an X: NEEDS COMMUNITY SUPPORT	
	- Not in need.<br>X Not satisfied<br>V Satisfied and name of the person involved in the network<br><br>Dates				10 INDEPENDENT<br>5 NEEDS SUPPORT<br>0 DEPENDENT				PERSON IDENTIFIED WITHIN THE CIRCLE OF THE COMMUNITY NETWORK: FULL NAME AND ROLE IN THE NETWORK.	CONTACT INFORMATION: PHONE NUMBER AND ADDRESS.
VISITS	V 0	V 1	V 2	V 3	V4	V 5	V6	V 7		
Ability to receive help										
Being satisfied by receiving it (0 – 10)										
Feeding:										
Bathing:										
Dressing:										
Grooming:										
Bowels (Incontinent)										
Bladder (Incontinent)										
Toilet Use unable to access or use										
Transfers (bed to chair and back) Needs two people.										
Mobility (on level surfaces)										
Use of stairs (up and down)										
Housekeeping										
Laundry										
Shopping										
Ability to use telephone										
Mode of transportation										
Ability to handle finances										
Responsibility for own medications										
Accompaniment in hospital or home or residential centre										
Management of visits to the consultation, emergency										
Accompaniment to medical visits										
Information										

management/ communication with healthcare professionals, family and decision-makers										
Entertainment activities										
Emotional release										
Final wishes										
Other needs:										
**Use and evaluation of the scales**	**V 0**	**V 1**	**V 2**	**V 3**	**V4**	**V 5**	**V6**	**V 7**		
TOTAL NEEDS	X	X	X	X	X	X	X			
TOTAL NETWORK OF CARE	X	X	X	X	X	X	X			
% COVERED NEEDS	X	X	X	X	X	X	X			
LONELINESS SCALE		X					X			
EUROQOL 5D		X					X			
USE OF THE HEALTHCARE SYSTEM		X					X			
ZARIT SCALE		X	X (X for the main caregiver, as new profiles appear)				X			
SCALE FOR NETWORK SATISFACTION								X		
