# Supplementary material for: Development and Management of Networks of Care at the End of Life (the REDCUIDA Intervention): Protocol for a Nonrandomized Controlled Trial
Source: JMIR Res Protoc. 2018 Oct 12;7(10):e10515. doi: 10.2196/10515 (PMC6231747; doi:10.2196/10515)
Supplement: Multimedia Appendix 3 [file resprot_v7i10e10515_app3.pdf]

APPENDIX 3. CIRCLE OF THE COMMUNITY NETWORK

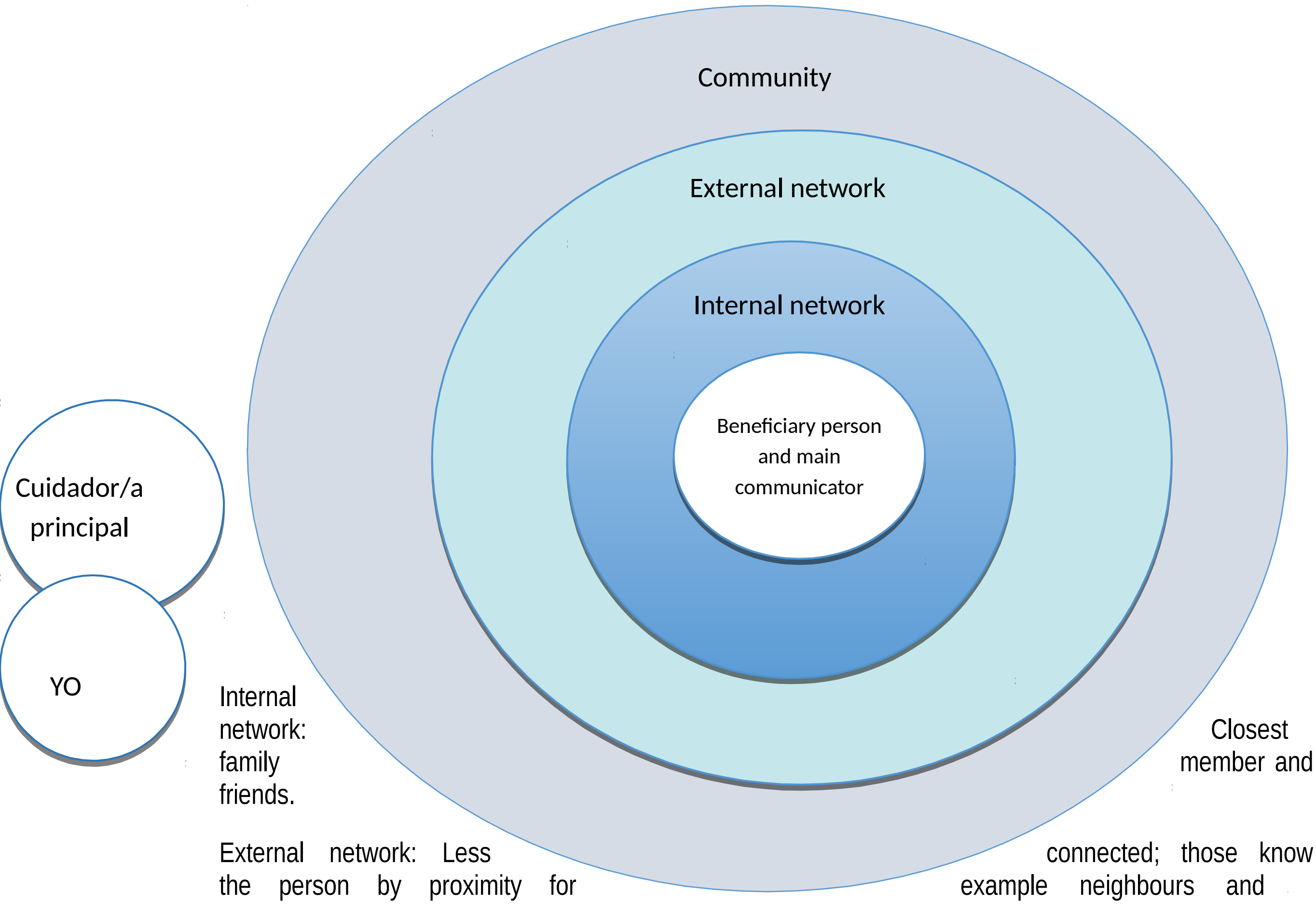

Internal [illegible] Closest
network: [illegible] member and
family
friends.

External network: Less [illegible] connected; those know
the person by proximity for [illegible] example neighbours and [illegible]
acquaintances.

Community: Volunteers, professionals (family doctors, social worker, advisor, psychologist, etc.)

Adapted from J.Abel’s Circles of Care.
