# Supplementary material for: Development and Management of Networks of Care at the End of Life (the REDCUIDA Intervention): Protocol for a Nonrandomized Controlled Trial
Source: JMIR Res Protoc. 2018 Oct 12;7(10):e10515. doi: 10.2196/10515 (PMC6231747; doi:10.2196/10515)
Supplement: Multimedia Appendix 4 [file resprot_v7i10e10515_app4.pdf]

APPENDIX 4. ESTE II LONELINESS SCALE.

Factor 1. Perception of social support			
	ALWAYS	SOMETIMES	NEVER
o Do you have anyone you can speak to about your problems?	0	1	2
o Do you believe there are people caring for you?	0	1	2
o Do you have friends and family when you need them?	0	1	2
o Do you feel heard?	2	1	0
o Do you feel sad?	2	1	0
o Do you feel lonely?	2	1	0
o At night do you feel alone?	2	1	0
o Do you feel loved?	0	1	2
Factor 2. Use of new technologies			
o Do you use a mobile phone?	0	1	2
o Do you use a computer (video games, memory games, etc.)?	0	1	2
o Do you use the internet?	0	1	2
Factor 3. Index for subjective social participation			
o During the week and on the weekends, do people call you to go out?	0	1	2
o Do you find it easy to make friends?	0	1	2
o Do you go to any parks or pensioner's homes where you can interact with other elderly people?	0	1	2
o Do you like participating in leisure activities that are organised in your town/community?	0	1	2
POINTS SCORED FOR SOCIAL LONLINESS ____________________Points			

Three levels of social loneliness can be identified based on this scoring: low, medium and high (table 2)

**Levels of points on the ESTE II scale: SOCIAL LONELINESS**	
**LEVELS**	**SCORE**
**Low**	0 a 10 points
**Medium**	11 a 20 points
**High**	21 a 30 points
