# Supplementary material for: Development and Management of Networks of Care at the End of Life (the REDCUIDA Intervention): Protocol for a Nonrandomized Controlled Trial
Source: JMIR Res Protoc. 2018 Oct 12;7(10):e10515. doi: 10.2196/10515 (PMC6231747; doi:10.2196/10515)
Supplement: Multimedia Appendix 5 [file resprot_v7i10e10515_app5.pdf]

APPENDIX 5. EUROQOL 5D QUALITY OF LIFE SCALE

Please mark an X in the box next to the statement that best described your health status today.

Mobility
- I do not have problems walking.
- I have some problems walking.
- I am bedridden.

Personal care
- I have no problems for personal care.
- I have some trouble washing or dressing myself.
- I am unable to wash or dress myself.

Daily activities (e.g. working, studying, chores, family activities during spare time)
- I do not have problems conducting my daily activities
- I have some problems conducting my daily activities
- I am unable to conduct my daily activities

Pain/ Discomfort
- I do not have any pain or discomfort
- I have moderate pain or discomfort
- I have a lot of pain or discomfort

Anxiety/Depression
- I am not anxious or depressed
- I am moderately anxious or depressed
- I am very anxious or depressed

The best health state imaginable

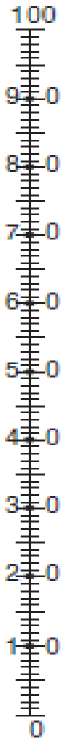

The worst health state imaginable

VERTICAL VISUAL ANALOGUE SCALE FOR SELF-RATED HEALTH

To help people describe how good or bad your state of health is this scale has been drawn in a similar way to a thermometer, showing the best imagined health status at the top and the worst health state imaginable at the bottom.

Please indicate your opinion, on this scale, of how good or bad your state of health is on today. To do so, draw a line from the box that says "Your state of health today" (seen below) to the point of the thermometer that in your opinion indicates how good or bad you rate it.

Your health status today
