# Supplementary material for: Development and Management of Networks of Care at the End of Life (the REDCUIDA Intervention): Protocol for a Nonrandomized Controlled Trial
Source: JMIR Res Protoc. 2018 Oct 12;7(10):e10515. doi: 10.2196/10515 (PMC6231747; doi:10.2196/10515)
Supplement: Multimedia Appendix 6 [file resprot_v7i10e10515_app6.pdf]

APENDIX 6. ZARIT SCALE FOR CAREGIVER BURDEN.

Zarit scale for caregiver burden (Interview to assess caregiver burden)

	Score
1. Do you feel that, due to the time you have dedicated to your family member, you do not have enough time for you?	
2. Do you feel stressed between caring for your relative and trying to meet other responsibilities for your family or work?	
3. Do you feel that your relative current affects your relationships with other family members or friends in a negative way?	
4. Do you feel tired when you are around your relative?	
5. Do you feel your health has suffered because of your involvement with your relative?	
6. Do you feel you have lost control of your life since your relative's illness?	
7. Overall, how burdened do you feel in caring for your relative?	

Each item is evaluated as follows:

**Frequency**	**Score**
Never	0
Rarely	1
Sometimes	2
Quite frequently	3
Nearly always	4

You must add the point rated for each of the values to obtain the final score.

There are not rigid scopes established, though a score between 17 points is considered as not a burden and those over 17 points is considered to be a severe burden.
