# Supplementary material for: Development and Management of Networks of Care at the End of Life (the REDCUIDA Intervention): Protocol for a Nonrandomized Controlled Trial
Source: JMIR Res Protoc. 2018 Oct 12;7(10):e10515. doi: 10.2196/10515 (PMC6231747; doi:10.2196/10515)
Supplement: Multimedia Appendix 7 [file resprot_v7i10e10515_app7.pdf]

APPENDIX 7. FAMILY AND SOCIAL SUPPORT NETWORK SCALE OF SATISFACTION

Name:
Age:
Gender:

Professional reference:

Relationship with the patient:

- o Spouse
- o Son/Daughter
- o Sibling
- o Parent
- o Niece/nephew
- o Grandchild
- o Other family relationship: Please specify
- o Professional caregiver
- o Other: Please specify

1. Do you believe that the patient's needs were met by the community promoter?

o Yes o No o Somewhat o Unsure/No reply o

2. Do you believe that the identified needs were met with the support of the community?

o Yes o No o Somewhat o Unsure/No reply o

3. Have you felt satisfied with the support of the care network?

o Yes o No o Somewhat o Unsure/No reply o

4. Did the community promoter who attended you teach or advise you on the best way to create networks to take care of you and accompany you?

o Yes o No o Somewhat o Unsure/No reply o

5. Was the promoter of the community friendly while involved?

o Yes o No o Somewhat o Unsure/No reply o

6. When you called the community promoter or a member of the palliative care team, did their solve your issue or resolver your questions?

o Yes o No o Somewhat o Unsure/No reply o

7. Did the community promoter provide you with resources that would allow you to meet the patient's needs? (Volunteering, catering, etc).

o Yes o No o Somewhat o Unsure/No reply o

8. Are you satisfied with the participation in All with you programme?

o Yes o No o Somewhat o Unsure/No reply o

9. Please evaluate the overall involvement of the community promoter. On this scale 1 is very bad and 10 is very good. How do you rate your experience?

Very bad 1 2 3 4 5 6 7 8 9 10 Very good

10. Comments and suggestions
