# Supplementary material for: Development and Management of Networks of Care at the End of Life (the REDCUIDA Intervention): Protocol for a Nonrandomized Controlled Trial
Source: JMIR Res Protoc. 2018 Oct 12;7(10):e10515. doi: 10.2196/10515 (PMC6231747; doi:10.2196/10515)
Supplement: Multimedia Appendix 8 [file resprot_v7i10e10515_app8.pdf]

APPENDIX 8. SCALE FOR THE USE OF THE HEALTHCARE SYSTEM

A questionnaire for the beneficiary (to be completed by the community promoter)
In the final month:

How many times did you visit your family doctor or nurse?	
Did a family member accompany you?	
How long were these visits with the family doctor or nurse (in minutes)?	
How many times did you visit the specialist doctor or nurse?	
Did a family member accompany you?	
How long were these visits with the specialist doctor or nurse (in minutes)?	
